# Supplementary material for: AffyMAPSDetector: a software tool to characterize Affymetrix GeneChip™ expression arrays with respect to SNPs
Source: BMC Bioinformatics. 2007 Jul 30;8:276. doi: 10.1186/1471-2105-8-276 (PMC1959249; doi:10.1186/1471-2105-8-276)
Supplement: Additional file 13 — AffyMAPSDetector v1 source code. AffyMAPSDetector source code. [file 1471-2105-8-276-S13.zip › AffyMAPSDetector_v1_src/doc/index-files/index-7.html]

G-Index


|  |  |  |  |
| --- | --- | --- | --- |
| **Overview** \| Package \| Class \| Use \| **Tree** \| **Deprecated** \| **Index** \| **Help** | | |  |
| **PREV LETTER**   **NEXT LETTER** | **FRAMES**    **NO FRAMES**     **All Classes** |  |  |


A B C D E F G H I J L M N O P R S T V W X Y 

---


## **G**

**G** - Static variable in class affychip.snpdetector.util.PmMmHeatMapViewer: **GENE\_ANNOT** - Static variable in class affychip.snpdetector.Constant: **GENE\_ANNOT\_FIELD\_INDEX** - Static variable in class affychip.snpdetector.Constant: **GENE\_IDENTIFIER\_FIELD\_INDEX** - Static variable in class affychip.snpdetector.Constant: **GENE\_INFO\_FILE** - Static variable in class affychip.snpdetector.Constant: **GENE\_INFO\_FILE\_SEPARATOR** - Static variable in class affychip.snpdetector.Constant: **GENE\_INFO\_HEADERS** - Static variable in class affychip.snpdetector.Constant: **GENE\_INFO\_PROBESET\_ID\_FIELD\_INDEX** - Static variable in class affychip.snpdetector.Constant: **GeneAnnotLoaderWizard** - class affychip.snpdetector.util.GeneAnnotLoaderWizard.: **GeneInfo** - class affychip.snpdetector.model.GeneInfo.: Title: Probe SNP Detector **GeneInfo(String, String, String)** - Constructor for class affychip.snpdetector.model.GeneInfo: **GuiUtils** - class affychip.snpdetector.util.GuiUtils.: Title: Probe SNP Detector **GuiUtils()** - Constructor for class affychip.snpdetector.util.GuiUtils: **genBankAcc** - Static variable in class affychip.snpdetector.model.OptimizedSnpDetector: **getAffyMapsDetectorFrame()** - Method in class affychip.snpdetector.ui.AffyMapsDetectorUI: **getAlleles()** - Method in class affychip.snpdetector.model.SnpInfo: **getAttribute(Object)** - Method in class jwf.WizardContext: Gets an attribute. **getAxisOrientation()** - Method in class affychip.snpdetector.model.SnpInfo: **getColor(double)** - Method in class affychip.snpdetector.util.ColorGradient: **getCrossHbCheckFrame()** - Method in class affychip.snpdetector.ui.AffyMapsDetectorUI: **getDescription()** - Method in class affychip.snpdetector.model.GeneInfo: **getFileChooser()** - Static method in class affychip.snpdetector.util.PmMmHeatMapViewer: **getFixedWidth(String)** - Static method in class jwf.WizardUtils: **getFixedWidth(String, int)** - Static method in class jwf.WizardUtils: **getFunction()** - Method in class affychip.snpdetector.model.SnpInfo: **getGenBankAccNo()** - Method in class affychip.snpdetector.model.GeneInfo: **getGeneAnnot()** - Method in class affychip.snpdetector.util.PmMmMatrixLoader: **getGeneIdentifierFieldIndex()** - Method in class affychip.snpdetector.model.DataState: **getGeneInfo()** - Method in class affychip.snpdetector.model.DataState: **getGeneInfoFieldSeparator()** - Method in class affychip.snpdetector.model.DataState: **getGeneInfoFile()** - Method in class affychip.snpdetector.model.DataState: **getGeneInfoHeaders()** - Method in class affychip.snpdetector.model.DataState: **getGeneInfoMap()** - Method in class affychip.snpdetector.model.DataState: **getGeneInfoProbeSetIdFieldIndex()** - Method in class affychip.snpdetector.model.DataState: **getHasSnpsFilename()** - Method in class affychip.snpdetector.model.DataState: **getHeader()** - Static method in class affychip.snpdetector.model.GeneInfo: **getHeader()** - Static method in class affychip.snpdetector.model.SnpInfo: **getHeteroZygosity()** - Method in class affychip.snpdetector.model.SnpInfo: **getInstance()** - Static method in class affychip.snpdetector.model.DataState: **getInterrogationPosition()** - Method in class affychip.snpdetector.model.Probe: **getKey()** - Method in class affychip.snpdetector.model.Probe: **getLocusId()** - Method in class affychip.snpdetector.model.GeneInfo: **getLocusLinkFieldIndex()** - Method in class affychip.snpdetector.model.DataState: **getMax()** - Method in class affychip.snpdetector.model.Probe: **getMessage()** - Method in class affychip.snpdetector.model.event.StatusEvent: **getMiddlePosition()** - Method in class affychip.snpdetector.model.Probe: **getMin()** - Method in class affychip.snpdetector.model.Probe: **getMmPosFieldIndex()** - Method in class affychip.snpdetector.model.DataState: **getNoLocusLinkFilename()** - Method in class affychip.snpdetector.model.DataState: **getNoSnpsFilename()** - Method in class affychip.snpdetector.model.DataState: **getNucleotideAccNo()** - Method in class affychip.snpdetector.model.SnpInfo: **getOrganism()** - Method in class affychip.snpdetector.model.DataState: **getProbeFor(String, String)** - Method in class affychip.snpdetector.model.ProbeSet: **getProbeInfoFieldSeparator()** - Method in class affychip.snpdetector.model.DataState: **getProbeInfoFile()** - Method in class affychip.snpdetector.model.DataState: **getProbeInfoHeaders()** - Method in class affychip.snpdetector.model.DataState: **getProbeInfoMap()** - Method in class affychip.snpdetector.model.DataState: **getProbeInfoProbeSetIdFieldIndex()** - Method in class affychip.snpdetector.model.DataState: **getProbeSeqFieldIndex()** - Method in class affychip.snpdetector.model.DataState: **getProbeSet()** - Method in class affychip.snpdetector.model.DataState: **getProbesetId()** - Method in class affychip.snpdetector.model.GeneInfo: **getProbsetId()** - Method in class affychip.snpdetector.model.Probe: **getProtAccNo()** - Method in class affychip.snpdetector.model.SnpInfo: **getRange()** - Method in class affychip.snpdetector.model.Probe: **getRefSeqAccNo()** - Method in class affychip.snpdetector.model.GeneInfo: **getRsClusterId()** - Method in class affychip.snpdetector.model.SnpInfo: **getSeq()** - Method in class affychip.snpdetector.model.Probe: **getSequence()** - Method in class affychip.snpdetector.model.GeneInfo: **getSnpClass()** - Method in class affychip.snpdetector.model.SnpInfo: **getSnpInfoFor(String)** - Method in class affychip.snpdetector.model.GeneInfo: This method return the `SnpInfo` object for a given rs-cluster number. **getSnpInfos()** - Method in class affychip.snpdetector.model.GeneInfo: **getSnpList()** - Method in class affychip.snpdetector.model.Probe: **getSnpLoc()** - Method in class affychip.snpdetector.model.SnpInfo: **getSnps()** - Method in class affychip.snpdetector.model.GeneInfo: **getVisualArtifact()** - Method in class affychip.snpdetector.model.event.StatusEvent: **getWebGeneInfoFilename()** - Method in class affychip.snpdetector.model.DataState: **getWebSnpInfoFilename()** - Method in class affychip.snpdetector.model.DataState: **getWizardContext()** - Method in class jwf.WizardPanel: Get the wizard context. **getX()** - Method in class affychip.snpdetector.model.Probe: **getXPosFieldIndex()** - Method in class affychip.snpdetector.model.DataState: **getY()** - Method in class affychip.snpdetector.model.Probe: **getYPosFieldIndex()** - Method in class affychip.snpdetector.model.DataState

---


|  |  |  |  |
| --- | --- | --- | --- |
| **Overview** \| Package \| Class \| Use \| **Tree** \| **Deprecated** \| **Index** \| **Help** | | |  |
| **PREV LETTER**   **NEXT LETTER** | **FRAMES**    **NO FRAMES**     **All Classes** |  |  |


A B C D E F G H I J L M N O P R S T V W X Y 

---
